# Celery‐derived scaffolds with liver lobule‐mimicking structures for tissue engineering transplantation

**DOI:** 10.1002/SMMD.20220002

**Published:** 2022-12-16

**Authors:** Jinglin Wang, Xueqian Qin, Bin Kong, Haozhen Ren

**Affiliations:** ^1^ Department of Hepatobiliary Surgery Nanjing Drum Tower Hospital Clinical College of Traditional Chinese and Western Medicine School of Pharmacy Nanjing University of Chinese Medicine Nanjing Jiangsu China; ^2^ State Key Laboratory of Bioelectronics School of Biological Science and Medical Engineering Southeast University Nanjing China

**Keywords:** bio‐mimic, decellularized scaffold, liver regeneration, liver transplantation, tissue engineering

## Abstract

Decellularized scaffolds have a demonstrated value in liver tissue engineering. Challenges in this area are focused on effectively eliminating the biological rejection of scaffolds and finding a suitable liver cell source. Here, inspired by the natural microstructure of hepatic lobules, we present a novel decellularized celery‐derived scaffold cultured with human‐induced pluripotent stem cell‐derived hepatocytes (hiPSC‐Heps) bioengineering liver tissue construction. Because of the natural hollow channels, interconnected porous structures, and excellent physicochemical characterization of the decellularized celery‐derived scaffold, the resultant bioengineering liver tissue can maintain the hiPSC‐Heps viability and the hepatic functions in the in vitro cultures. Based on this bioengineering liver tissue, we have demonstrated its good biocompatibility and the significantly higher expressions of albumin (ALB) and periodic acid‐schiff stain (PAS) when it was implanted in nude mice. These remarkable properties endow the hiPSC‐Heps integrated decellularized celery scaffolds system with promising prospects in the field of liver transplantation and other regeneration medicine.

## Highlights

- A liver substitute via recellularizing the celery stem tissue was fabricated.
- The decellularized celery‐derived scaffold maintains cell viability and function.
- In vivo compatibility was also confirmed through specific ectopic transplantation.

## Graphical abstract

A novel decellularized celery‐derived scaffold with human induced pluripotent stem cell‐derived hepatocytes (hiPSC‐Heps) culture was fabricated for bioengineering liver tissue. The adequate hollow channels, interconnected porous structures of scaffolds, could promote hiPSC‐Heps to adhere and proliferate. In vivo studies were also performed to verify the high biocompatibility and special hepatic function.

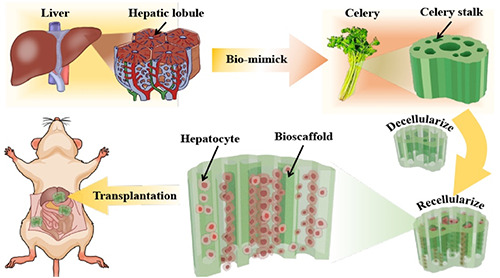

## 1 INTRODUCTION

Acute liver failure, caused by massive hepatocellular injury within a short period of time, is a series of severe clinical syndromes with high mortality.^1^, ^2^ Despite having diverse causes such as infection, hepatotoxic drugs, or immune‐mediated attack, acute liver failure is characterized by remarkably similar clinical features of severe liver impairment.^3^ Many therapeutic strategies aimed to treat simple complications and decelerate disease progression for liver regeneration have been developed and applied in clinic. However, most of these strategies are limited by the regenerative ability and long‐term recovery time of the liver. As an alternative, tissue engineering is recognized as one of the most promising techniques to solve this problem.^4^, ^5^, ^6^ Among different tissue engineering strategies, decellularized scaffolds have played an important role due to their advantages of maintaining cell functions and promoting the formation of new tissues in recent years.^7^, ^8^, ^9^ However, the decellularized scaffolds are principally derived from animal livers, which can lead to inevitable immune rejection due to species differences, while the scaffolds from other tissues lack the natural structure of the hepatic lobule. In addition, the availability of these scaffolds is usually limited, and the cells suitable for tissue‐engineered livers are usually in short supply or unavailable. Therefore, it is anticipated to develop easily available cell sources and biocompatible non‐animal decellularized scaffolds for tissue engineering liver.^10^, ^11^, ^12^

**FIGURE 1:**
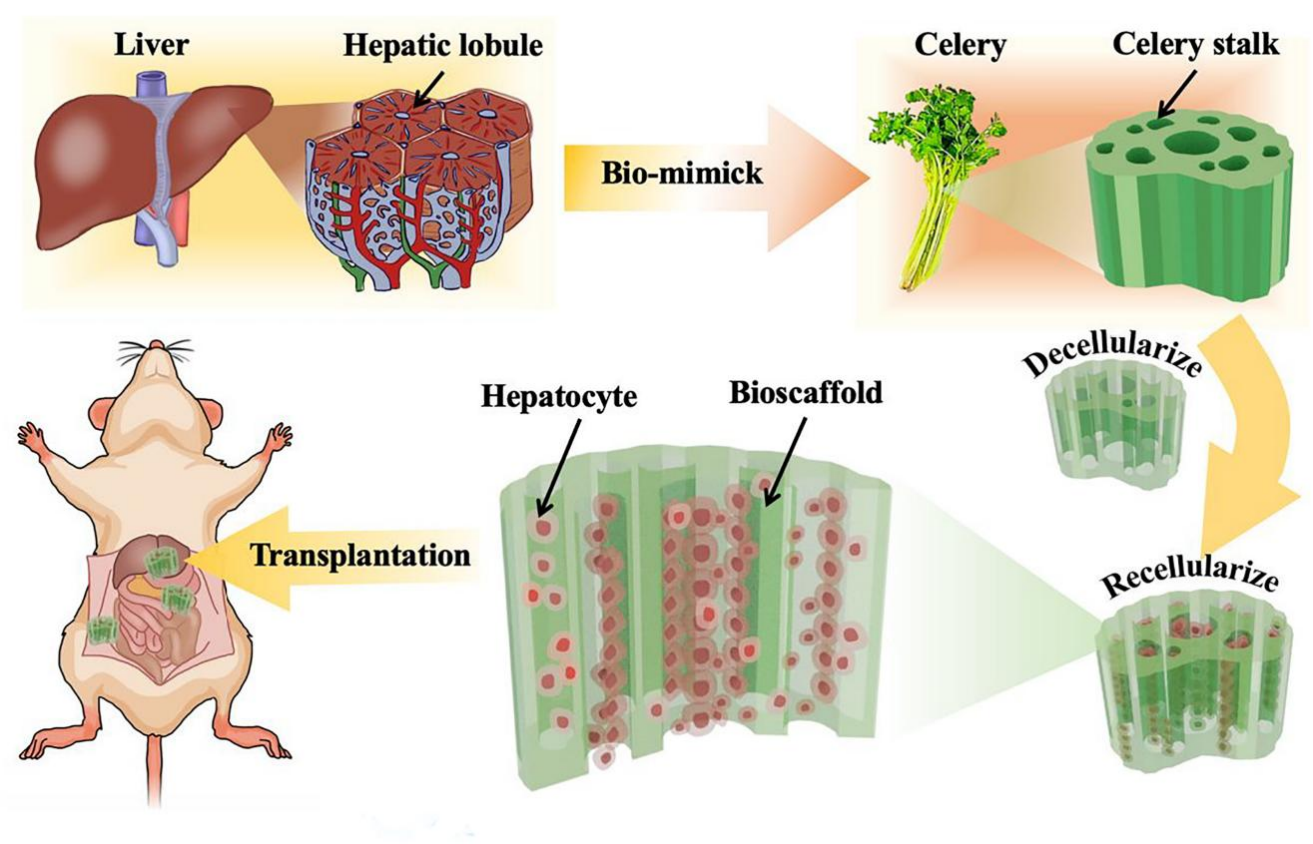
The schematic diagram of the decellularized celery stem applied as a bio‐mimicking 3D scaffold with hiPSC‐Heps for in vitro liver reconstruction and in vivo transplantation to the liver, spleen, and omentum of the nude mouse.

Here, inspired by the natural microstructure of hepatic lobules, we present a novel decellularized celery‐derived scaffold with human‐induced pluripotent stem cell‐derived hepatocytes (hiPSC‐Heps) culture for bioengineering liver tissue construction, as shown in Figure 1. In our liver, the hepatic lobule is composed of a central vein running through its center, surrounded with hepatocytes radiating in all directions, and clusters of five to seven vessels at its edges.^13^, ^14^ Coincidentally, the stem of cheap and readily available celery is a collection of many thin tubes with a big one at the center, which is similar to the structure of the hepatic lobule. Generally, with the decellularization of vegetable tissues, including the celery stem, to remove their cellular components and preserve their natural hierarchical structures, the resultant scaffolds are nontoxic and can allow cells to adhere and proliferate.^15^, ^16^, ^17^ In contrast, the hiPSCs have become a safe cell source for potential clinical applications because of the elimination of genomic integration and background transgene expression.^18^ They can replicate efficiently and have the capacity to differentiate into hepatocytes. Thus, if the decellularized celery scaffolds and the hiPSC‐Heps can be effectively integrated, a superior tissue‐engineered liver is expected to be developed.^4^, ^19^, ^20^

In this paper, we fabricated the decellularized celery scaffolds with hiPSC‐Heps culture for the desired bioengineering liver tissue construction. The decellularized celery stem has been characterized in terms of biocompatibility for liver tissue engineering through cell proliferation assay. It was found that due to their adequate hollow channels, interconnected porous structures,^21^ and excellent mechanical properties, the scaffolds could promote hiPSC‐Heps to adhere and proliferate. Besides, the scaffold culture system could maintain the morphologies and functions of hiPSC‐Heps for prolonged periods of time compared with the traditional two‐dimensional (2D) culture. Furthermore, in vivo studies were also performed by implanting the bioengineering liver tissue into the liver of nude mice. It was demonstrated that this hiPSC‐Heps integrated decellularized celery scaffold system was conductive to exhibit high biocompatibility and special hepatic function. To summarize, the suggested bioengineering liver tissues with decellularized celery scaffolds and hiPSC‐Heps definitely possess the ability to implant, thereby indicating their important value in acute liver failure treatment.

## 2 RESULTS AND DISCUSSION

**FIGURE 2:**
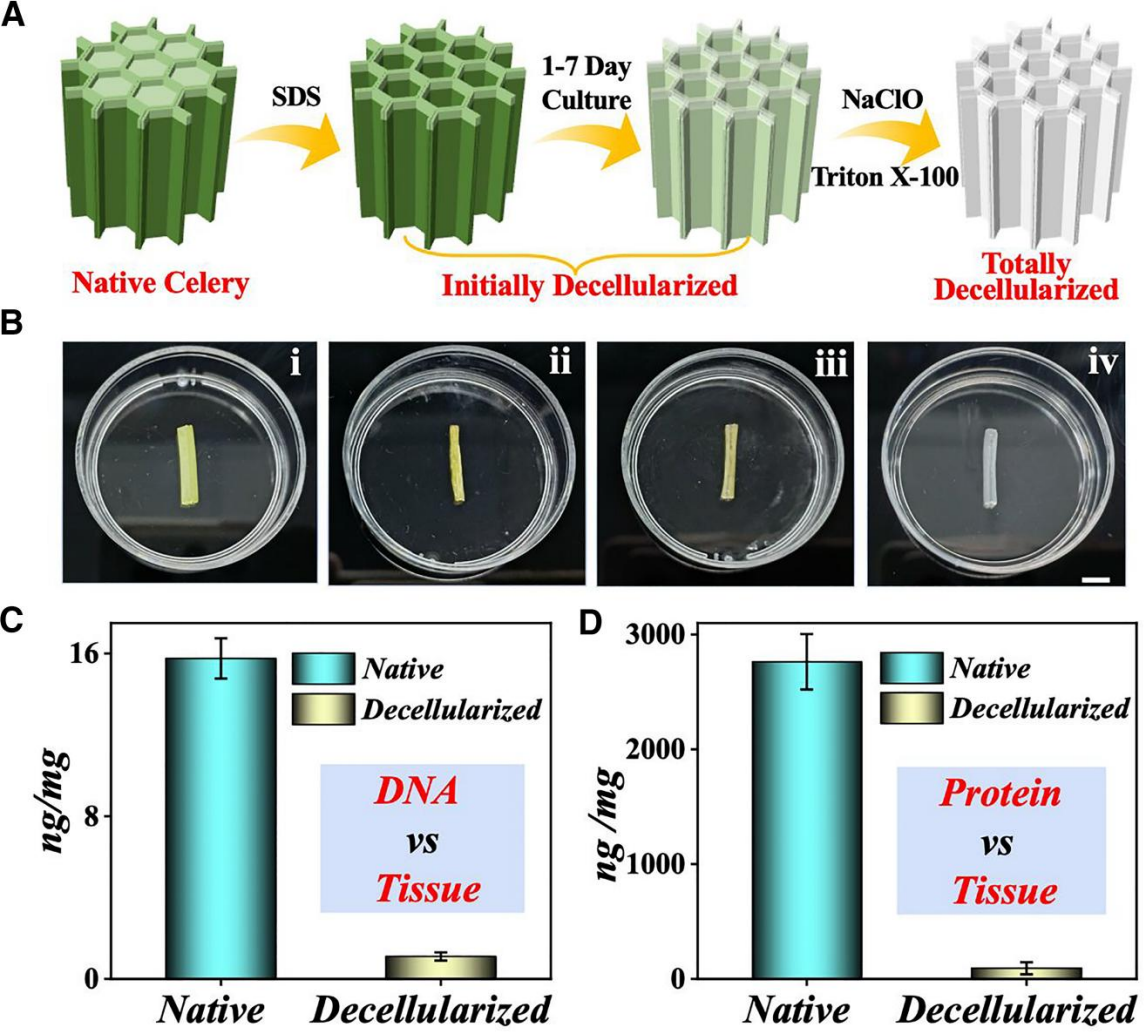
The schematics and characterization of the decellularization process of the celery stem. (A) The schematic illustration of the decellularization process of the natural celery. (B) The photographs of the celery stem during the decellularized process: (i) untreated fresh celery; (ii, iii) immersed in 10% SDS for 1 (ii) and 5 (iii) days, respectively; (iv) 2 days after soaking in 1% NaOCl containing 0.1% Triton X‐100. The scale bar is 0.5 cm. (C, D) The comparison of DNA content (C) and protein content (D) between natural celery tissue and decellularized celery scaffold.

In a typical experiment, the bio‐mimicking 3D scaffold was developed from a section of the celery stem. To make the stem suitable for further functionalization, decellularization was required in processing the fresh substrate. Specifically, the celery stem was initially decellularized by immersing it in 10% sodium dodecyl sulfate (SDS) for 7 days and then treating it with 1% sodium hypochlorite (NaOCl) containing 0.1% Triton X‐100 for final decellularization (Figure 2A).^22^, ^23^ As illustrated in Figure 2B, the celery stem was bleached and turned sub‐transparent with time due to the elution of green plant cells. To validate the decellularization effect, the content of DNA and protein in the treated tissue composition was detected. As shown in Figure 2C,D, the decellularized scaffold contained almost no protein or DNA compared with the natural celery tissue, and the level of residual protein and DNA met the limits for decellularized tissue (<50 ng DNA/mg tissue), indicating that the decellularization process can effectively elute the protein and DNA from the fresh tissue. It is worth mentioning that the complete removal of protein and DNA from the celery stem can contribute to the reduction of immune rejection and improvement of biocompatibility when culturing hepatic cells.^24^

**FIGURE 3:**
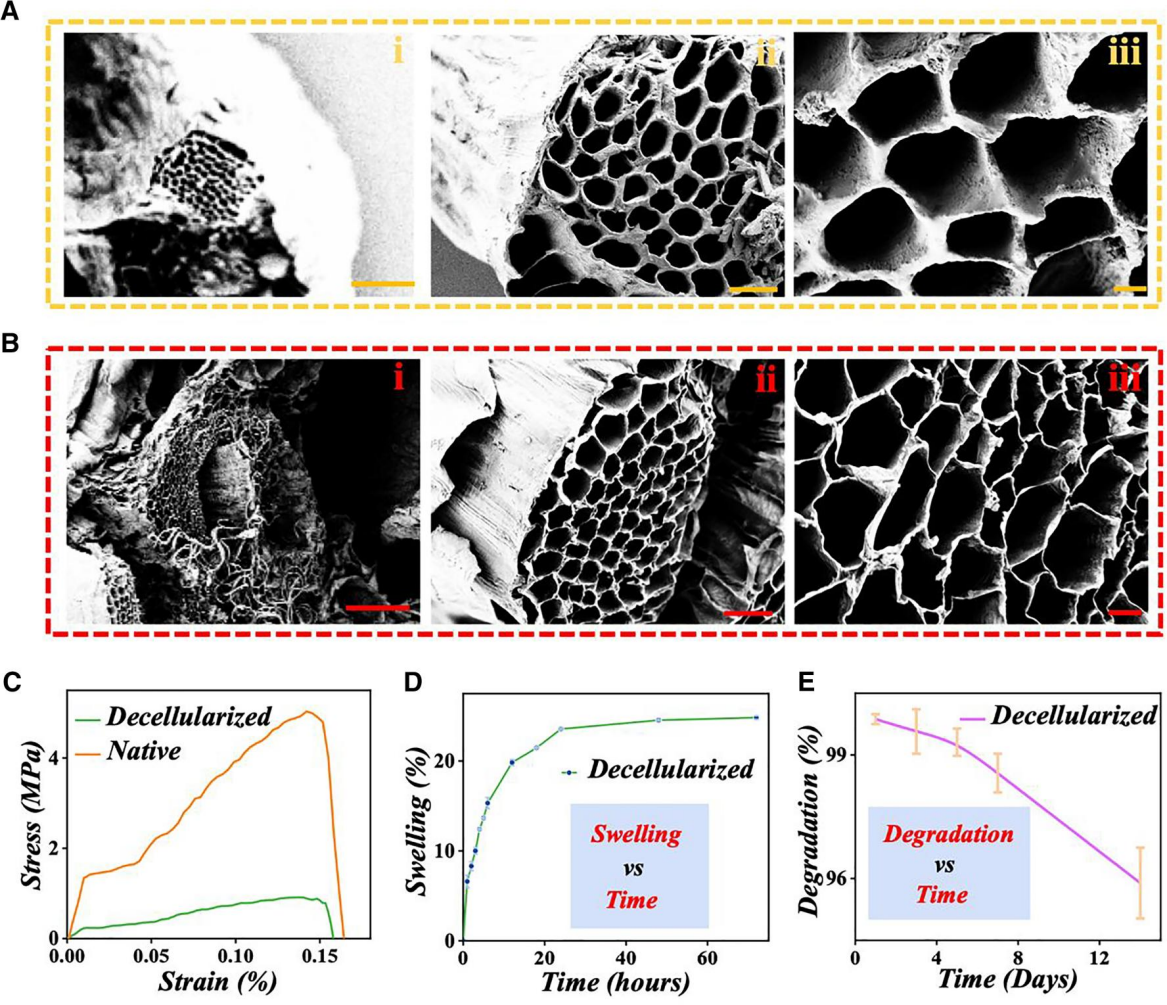
The SEM images and physicochemical functions of natural celery tissue and decellularized celery scaffold. (A) The SEM images of natural celery tissue. (B) The SEM images of decellularized celery tissue. The scale bars are 100 μm in (A(i)) and (B(i)), 20 μm in (A(ii)) and (B(ii)), and 5 μm in (A(iii)) and (B(iii)), respectively. (C) The strain‐stress curves of natural celery and decellularized celery. (D) The water absorption of the decellularized celery scaffold. (E) The degradation of the decellularized celery scaffold in PBS at 37°C for 14 days. SEM, scanning electron microscope.

The celery stem is known for its tightly arranged parallel hollow microstructures, which provides an ideal substrate and foundation for the tissue reconstruction. To demonstrate this feature, the microstructures of the fresh celery stem and decellularized celery stem were characterized by the scanning electron microscope (SEM) (Figure 3A,B). As shown in Figure 3A(i–iii), the celery stem demonstrated an obvious tightly packed hollow microstructure, which was very similar to the microstructures of liver lobules. Identically, the decellularized celery stem maintained the specific microstructure showing the integrated porous morphology with thinner walls and larger apertures (Figure 3B(i–iii)), which indicated that the decellularization of celery would not damage the inner microarchitectures that could provide suitable spaces for cell landing. The diameters of the hepatocytes range from 12 to 40 μm, as shown in the SEM pictures in Figure 3A,B which showed a pore size of the scaffold ranging from 20 to 150 μm. The adequate size of the decellularized celery‐derived scaffold was suitable for hepatocytes' proliferation and migration. Besides, the mechanical properties of the fresh and the decellularized celery stem scaffolds were also compared. As illustrated in Figures 3C, S1 and S2, the tensile stress under external pulling force of the natural celery stem tissue reached 2.96 ± 1.08 MPa when stretched to 1.10 ± 0.03 times, while that of the decellularized tissue was 0.81 ± 0.18 MPa when stretched to 1.16 ± 0.04 times. This result demonstrated that the decellularized scaffold was flexible enough to support cell adhesion.^25^ Compared to the directly lyophilized protein scaffolds, celery‐derived decellularized scaffold exhibited advantages in low production cost, abundant supplies, simple operation, and suitability for research. Next, we elucidated the water‐absorbent property of the decellularized celery stem. When the decellularized stem was immersed in phosphate buffer saline (PBS) at 37°C over time, its whole weight gradually increased, indicating the excellent hydrophilicity and water absorbability of the scaffold (Figure 3D). Thus, it could be inferred that when the scaffold was applied to cell culture and tissue reconstruction, it could effectively absorb culture media and nutrients from the surroundings, thus providing the humoral environment where cells can proliferate well. Later, the stability of the scaffold was evaluated by placing the decellularized celery stem in PBS at 37°C for 14 days. As shown in Figure 3E, no obvious degradation was observed in the scaffold, which demonstrated the favorable stability of the decellularized tissue for culturing cells.^26^, ^27^

**FIGURE 4:**
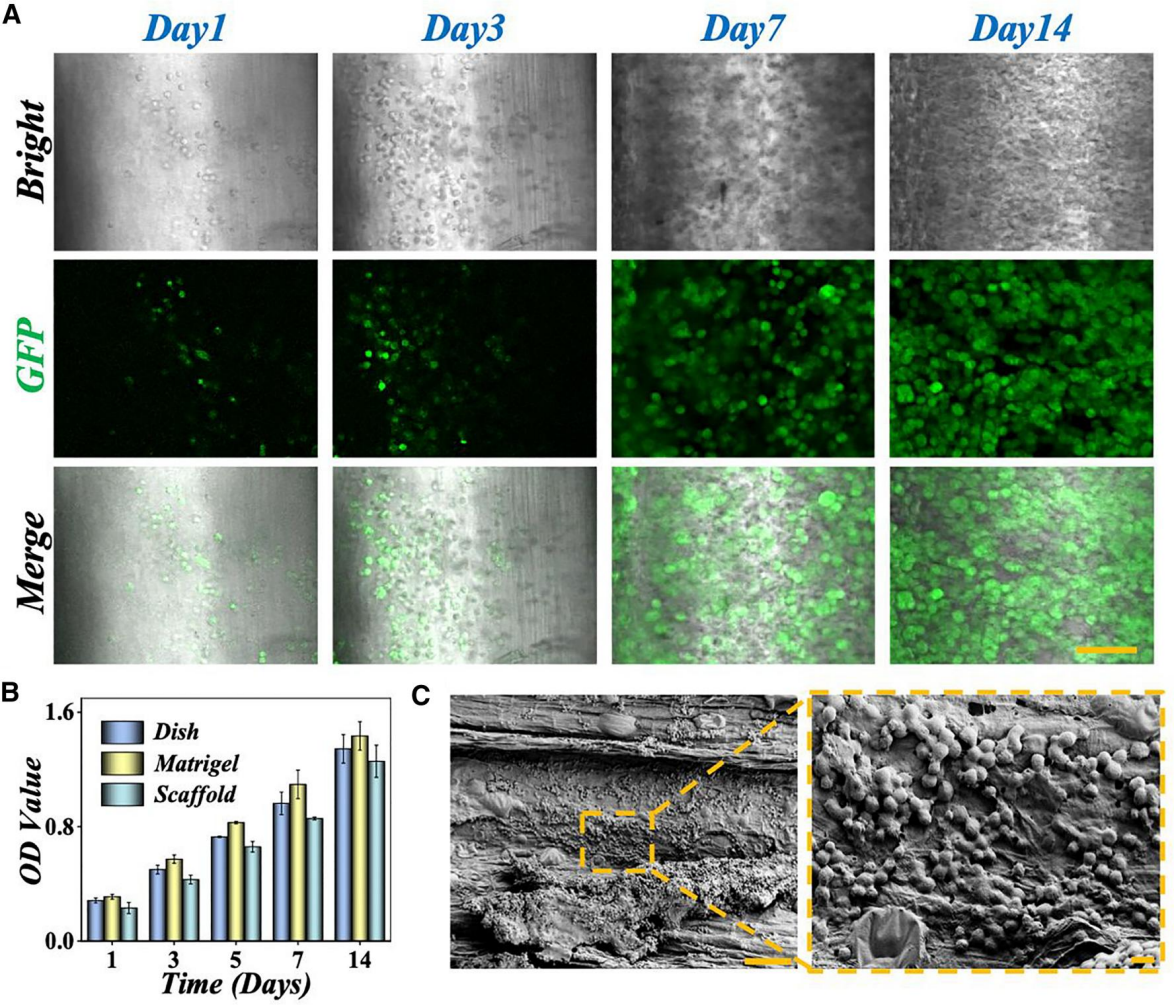
(A) The confocal laser scanning fluorescence images of hiPSC‐Heps cultured on decellularized celery scaffolds for 14 days. The scale bar is 100 μm. (B) The CCK‐8 results of cells cultured in conventional 2D dish, matrigel, and decellularized celery stem scaffold. (C) The SEM images of hiPSC‐Heps adhered on the scaffold. The scale bars are 200 μm (left) and 20 μm (right), respectively.

Since the decellularization process involved many necessary detergents, enzymes, ion chelators, and acids/bases, which could result in cytotoxicity if residues were not totally removed, the biocompatibility of the prepared scaffold should be confirmed before further applied to tissue reconstruction. Notably, to assess the feasibility of decellularized scaffold as a substrate for cell culture, the hiPSC‐Heps were selected as an optimal cell source for the biocompatibility test. The hiPSC‐Heps can detoxicate ammonia, synthesize glycogen, secrete albumin and urea, express key CYP450 proteins and pivotal hepatic transcription factors and liver functional genes, which make their functions highly compatible to primary human hepatocytes.^18^ ^,^ ^28^ To specifically examine the biocompatibility of the scaffolds, the hiPSC‐Heps embedded in matrigel were infused into the decellularized celery scaffold and cultured for 14 days, while the cells with/without matrigel seeded in the 24‐well plate served as the control groups. As shown in Figure 4A, the viability of cells were determined by using GFP staining. On the third day after seeding, the number of GFP‐positive cells was obviously higher than that on the first day. Additionally, the number of GFP‐positive cells increased with the extension of culture time, suggesting that the cells could grow and proliferate well in the decellularized celery stem scaffold. Moreover, the cell counting kit‐8 (CCK‐8) test also demonstrated a corresponding result. As shown in Figure 4B, the viability of hiPSC‐Heps increased both in groups of conventional 2D dish, matrigel, and decellularized celery scaffold with culture duration from 1 to 14 days. These results verified the favorable biocompatibility of our prepared celery stem scaffold.

Furthermore, the matrigel used in this study was immobilized in the scaffolds, which was conducive to the formation of hiPSC‐Hep cell spheroids. Many studies have shown that hepatocytes would gradually lose liver‐specific functions within a few days when cultured in 2D monolayer culture conditions. Thus, the 3D spheroid formation is desirable because it is similar to the tight cell–cell interaction in the real liver and inherits better functions.^29^ As shown in Figure 4C, the hiPSC‐Heps formed small 3D aggregates in the scaffolds with a high density of single cells and cell–cell contact with tight junctions. In addition, from the enlarged view of the SEM image, the condition of the hiPSC‐Hep aggregates was even more clearly, in which the extending pseudopodia were observed between the cells, further facilitating the cells to attach tightly to the inner surface of the scaffold. As mentioned earlier, the decellularized scaffold effectively increased the contact area between the cells and matrix and also provided a larger and 3D microenvironment for better cell growth.

**FIGURE 5:**
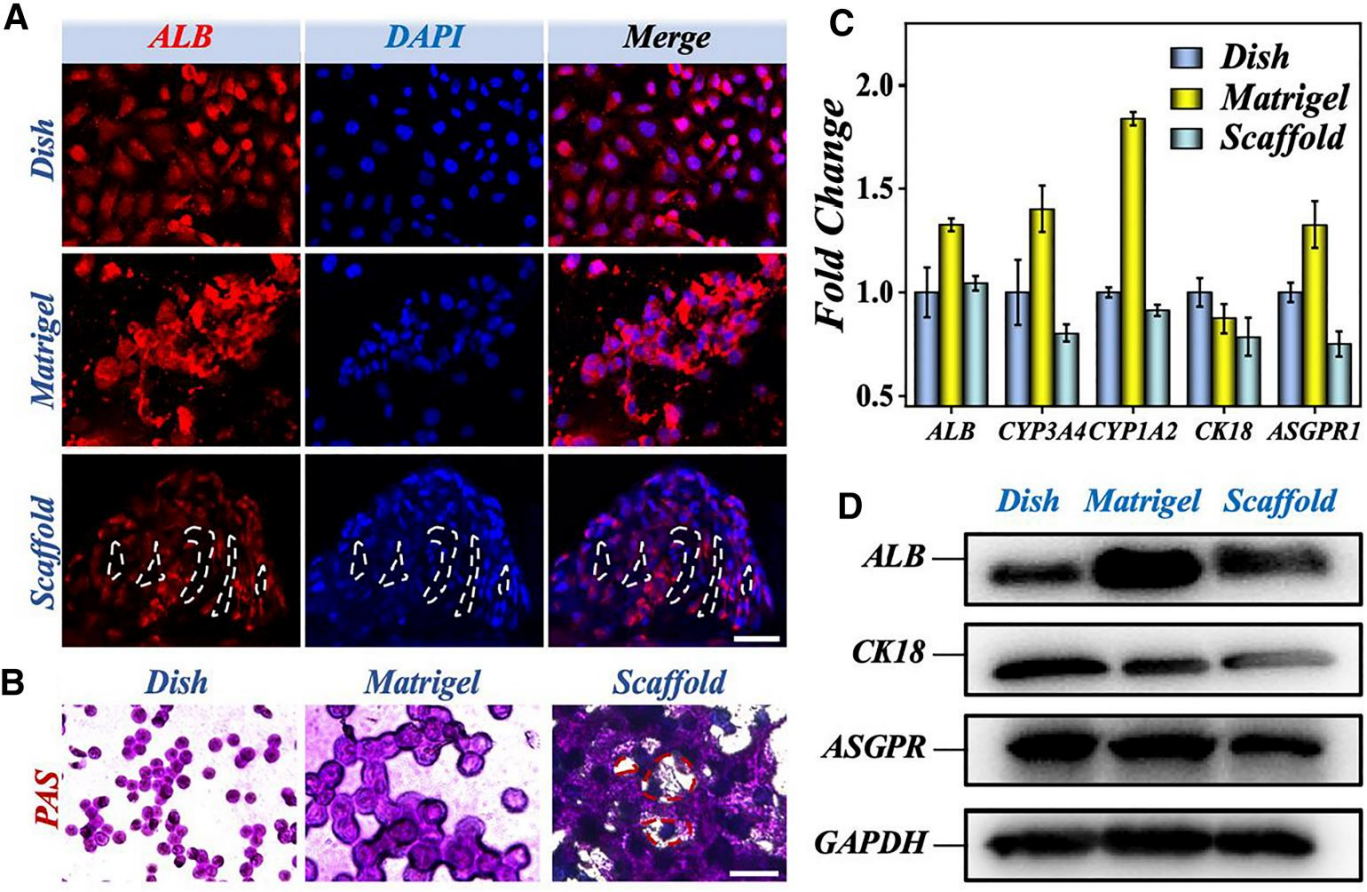
The hepatic special function of hiPSC‐Heps adhered on the scaffold. (A) The fluorescence images of the immunofluorescence level of ALB (red) in hiPSC‐Heps adhered on 2D conventional dish, matrigel, and decellularized celery scaffold. The nucleus was stained blue through DAPI staining. The scale bar is 50 μm. (B) The PAS staining of hiPSC‐Heps in conventional 2D dish, matrigel, and decellularized celery scaffold. The scale bar is 50 μm. (C) The RNA expression of hepatic function factors of hiPSC‐Heps in conventional 2D dish, matrigel, and decellularized celery scaffold. (D) The WB results of ALB, CK18, and ASGPR expression in hiPSC‐Heps in conventional 2D dish, matrigel, and decellularized celery scaffold. ALB, albumin; PAS, periodic acid‐schiff stain; WB, Western blotting.

As mentioned above, hepatocytes lose their differentiation state over time when cultured in a 2D dish due to the lack of extracellular matrix components and cell–cell interactions. However, the composition of matrigel is heterogeneous with over 1500 different proteins in its composition, including the most common proteins, such as laminin and type IV collagen. To evaluate the metabolic activity of hiPSC‐Heps grown in the decellularized liver scaffolds, the albumin secretion, glycogen synthesis, and mRNA expression levels of pivotal hepatic transcription factors and functional genes were quantified. As shown in Figure 5A, the fluorescence images of albumin verified that the number of albumin‐positive cells were larger in both matrigel and decellularized scaffold groups compared with the dish group, which were ascribed to the formation of hiPSC‐Heps 3D spheroids in the former two groups. In addition, the assessment of glycogen synthesis using periodic acid‐schiff stain (PAS) staining showed that the hiPSC‐Heps in each group expressed glycogen, indicating that the hiPSC‐Heps have primary hepatocyte function (Figures 5B, S3 and S4).

Hepatocyte‐specific gene expression was also studied during the culture process to determine the extent to which hiPSC‐Heps could maintain liver‐specific functions under different culture conditions over a long period of time. The genes detected in each group included ALB, Cytochrome P‐450 3A4 (CYP3A4), Cytochrome P‐450 1A2 (CYP1A2), Cytokeratin 18 (CK18), and Asialoglycoprotein receptor (ASGPR). As shown in Figure 5C, the expression of ALB in matrigel and decellularized scaffold groups was higher than that in the dish group, indicating their better function of liver cells. We further studied the mRNA expression levels of the crucial CYP enzymes CYP1A2 and CYP3A4, which affect CYP‐mediated drug metabolism and inhibition. The mRNA levels in the decellularized scaffold group were lower than those in the other two groups, and similar results were also obtained in CK18 and ASGPR1 tests. Western blotting (WB) results showed that the hiPSC‐Heps could express mature hepatocyte markers, such as ALB, CK18, and ASGR1, but there was no significant difference among the three groups (Figure 5D). This study demonstrated that the scaffold‐based 3D liver model had drug metabolism and detoxification ability. Since the 3D models allowed cells to mimic the in vivo microenvironment of appropriate cell–cell interactions, cell–matrix interactions, temporal and spatial biochemical gradients, the functional state of cells was significantly improved under this culture condition. Therefore, liver engineering structures constructed on the prepared scaffolds have a more stable liver phenotype.

**FIGURE 6:**
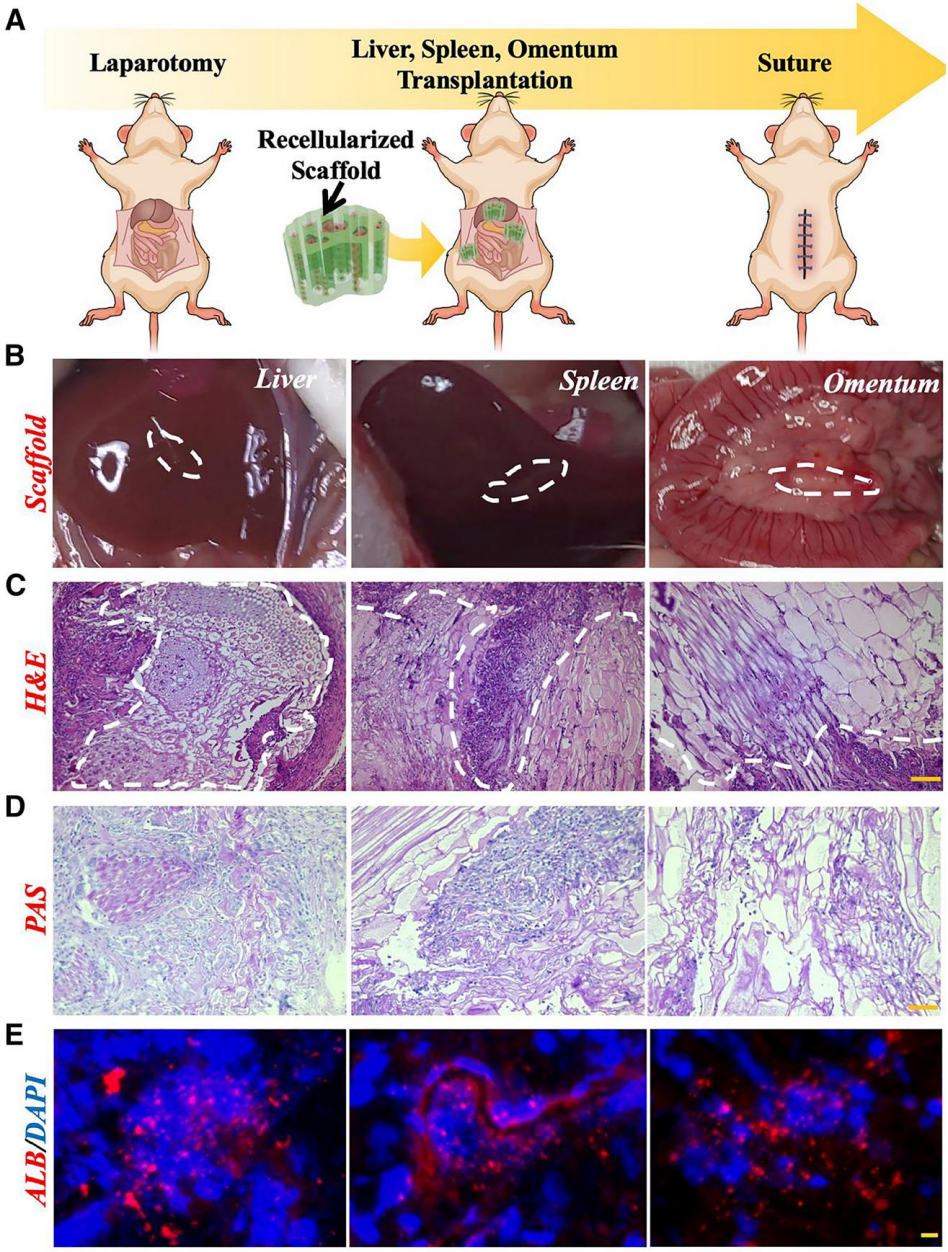
Animal study of the hiPSC‐Heps‐loaded decellularized celery stem scaffold transplantation. (A) Schematic illustration of the procedure of animal study designed to test the compatibility of hiPSC‐Heps‐loaded scaffold for liver, spleen, and omentum transplantation in a nude mouse model. (B) The photographs of the transplanted scaffolds wrapped in host tissues (liver, spleen, and omentum). (C) Histologic and (D) PAS immunohistochemical staining of hiPSC‐Heps in each group. Scale bars are 100 μm. (E) ALB (red) immunofluorescence staining of hiPSC‐Heps in each group. Scale bars are 10 μm. ALB, albumin; PAS, periodic acid‐schiff stain.

After verifying the biocompatibility and biofunctionality of the designed decellularized celery stem scaffold in vitro, its in vivo compatibility should also be evaluated, since it is an essential factor in estimating whether the scaffold is feasible for tissue engineering transplantation. As illustrated in Figure 6A, we prepared the hiPSC‐Heps‐loaded decellularized scaffolds for the ectopic transplantation on the liver, spleen, and omentum in nude mice. Two weeks after transplantation, the scaffolds were wrapped in the host tissues (liver, spleen, and omentum) and no inflammatory response was observed (Figure 6B). Histological staining of the omentum grafts showed normal liver cell morphology and the presence of vascular‐like structures at the center of the aperture compared with those at the liver and spleen (Figure 6C). It indicates that the graft has the ability to generate blood vessels. In addition, immunohistochemical staining of PAS and immunofluorescence staining of ALB also verified that the liver functions were successfully retained in the grafts, and hiPSC‐Heps from liver and spleen grafts also exhibited certain proliferation activity (Figure 6D,E). The above experimental results demonstrated that the designed scaffold possessed favorable in vivo compatibility and great potential in tissue engineering transplantation.

## 3 CONCLUSIONS

We developed an economic, highly bioactive, and sustainable liver substitutes by recellularizing the celery stem tissue with matrigel and hiPSC‐Heps. Through a serial decellularization process, the desired scaffold with a typical porous microstructure could be easily prepared. Because of the excellent biocompatibility of the decellularized plant tissue, the special microstructure of the celery stem, and the involvement of matrigel, the designed scaffold was suitable for cell culture and could maintain the cells in a 3D aggregation condition.^14^ This in vitro 3D model is able to support the growth and functionalization of the hepatocytes in a more effective way. Moreover, the in vivo compatibility of the celery stem‐derived scaffold was also confirmed through specific ectopic transplantation. Thus, this celery‐derived scaffold is very promising, which can be expected to have great prospects in tissue engineering transplantation.^30^, ^31^, ^32^

## 4 EXPERIMENTAL SECTION

Experimental details are provided in the Supporting Information.

## AUTHOR CONTRIBUTIONS

Jinglin Wang and Xueqian Qin completed the study and assembled data, Bin Kong and Jinglin Wang performed data analysis and wrote the manuscript. Haozhen Ren, Bin Kong, and Jinglin Wang conceived and designed the study, provided financial support and study materials, and gave final approval of the manuscript.

## CONFLICT OF INTEREST

The authors declare that they have no competing interests.

## ETHICS STATEMENT

This study was approved by the Committee on the Ethics of Animal Experiments of the Affiliated Drum Tower Hospital of Nanjing University Medical School (Approval No. 2018010017). All animal‐related protocols were authorized by the Institutional Animal Care and Use Committee of Nanjing University, China, which complied with the NIH Guide for the Care and Use of Laboratory Animals.

## Supporting information

Supporting Information S1

## Data Availability

All data needed to evaluate the conclusions in the paper are present in the paper and/or the Supplementary Materials. Additional data related to this paper may be requested from the authors.
