# Supplementary material for: Celery‐derived scaffolds with liver lobule‐mimicking structures for tissue engineering transplantation
Source: Smart Med. 2022 Dec 16;1(1):e20220002. doi: 10.1002/SMMD.20220002 (PMC11236025; doi:10.1002/SMMD.20220002)
Supplement: Supplementary file 1 — Supporting Information S1 [file SMMD-1-e20220002-s001.docx]

**Celery-derived scaffolds with liver lobule-mimicking structures for tissue engineering transplantation**

Jinglin Wang, Xueqian Qin, Bin Kong, Haozhen Ren *

**Experimental Section**

*Materials.* Celery was purchased from the local market. Sodium dodecyl sulphate(SDS), Trition-X-100 were bought from Biofroxx. 0.25% (w/v) trypsin-ethylene diamine tetraacetic acid (EDTA), BCA assay kit were purchased from KeyGEN BioTECH. Phosphate buffered solution (PBS) was bought from Bio-channel and fetal bovine serum (FBS) was bought from Gibco. DNA extraction kit was bought from BioFlux. ALB antibody was purchased from Abcepta. ASGR1 rabbit polyclonal antibody and CK18 polyclonal antibody were bought from Signalway Antibody. Superscript II Reverse Transcriptase Kit and Power SYBR Green PCR Master Mix were purchased from monad. CCK-8 kit, H&E and PAS kit were purchased from Solarbio.

*Decellularization of celery.* The celery stem was thoroughly washed with distilled water and cut into small pieces. The stem was then immersed in a solution of 10% SDS (w/v; SDS powder dissolved with PBS solution) in water for 5 days. Successively, the tissues were incubated in 0.1% Trition-X-100 (v/v; Trition-X-100 diluted with PBS solution) in a solution of bleach for 48 hours. The tissues were washed in an ultrasonic cleaner for 10 minutes and detergents were changed every day. The samples were then treated with PBS to eliminate the residues of detergents and lyophilized.

*Characterization of scaffold.* Decellularization of the celery was confirmed by quantification of DNA and protein content. The DNA and protein content in decellularized scaffold and native celery tissue were determined by isolating DNA and protein from each sample using a DNA extraction kit and BCA assay kit. The celery tissue before and after decellularization was dehydrated in a series of ethanol (50% to 100% for 15min each), sputter coated with gold and imaged using a scanning electron microscope for the evaluation of their morphological structure.

Native and decellularized celery stems were loaded into the grips of the mechanical testing machine to stretch using a force gauge with a 0.2 kg load cell (CDK HF-2). A stress–strain curve was obtained. Samples of decellularized scaffold were immersed in 1 mL PBS at room temperature to test its swelling ratio. At different intervals, samples were taken out and weighed after removing the residual water. The swelling ratio was calculated using the formula (W_s_-W_o_)/W_o_, where Wo and W_s_ were the original weight and swollen weight on respective time points, respectively. The swelling experiment was performed in triplicate.

*In vitro* degradation of the scaffolds was analyzed by immersing them in PBS (pH=7.4) at 37 °C for 1, 3, 5, 7 and 14 days. On respective time points, the samples were freeze-dried. Material remaining was evaluated using the formula, MR= 100-[(W_o_-W_t_)/Wo] × 100%, where W_o_ was the initial dry weight, and W_t_ was the dry weight, at a given time point. PBS was replaced with fresh buffer thrice in a week.

*Cell culture*. Utilize the differentiation strategy previously reported, the hiPSCs were first induced to differentiate into the hiPSC-Derived Hepatocytes (hiPSC-Heps). Then, the hiPSC-Heps was transfected with GFP lentivirus and cultured in 5% CO_2_ atmosphere at 37℃^[18]^. The celery scaffolds were sterilized in 75% ethanol and via ultraviolet irradiation for 6 h. To obtain cells-laden scaffold, 1.2×10^5^ hiPSC-Heps mixed well in every 10 μL microliter Matrigel were seeded on celery scaffolds. The cells-laden scaffolds were imaged with a scanning confocal microscope (Leica Microsystems, Buffalo Grove, IL) on days 1, 3, 5, 7, 14. The viability and growth rate of hiPSC-Heps were analyzed on days 1, 3, 5, 7, 14 by CCK-8 kit. Cells and cells mixed well in Matrigel on standard 24-well tissue culture plate were taken as 2D monolayer control for the study. The surface structures of the cell-laden scaffolds were characterized by SEM after the samples were immersed in 2.5% glutaraldehyde overnight and dehydrated in a series of ethanol (50% to 100% for 15min each).

*Quantitative Polymerase Chain Reaction.* We then analyzed the functional state of the cells cultured on the scaffold. For comparative studies, cells and cells mixed in Matrigel were seeded on the plate under the same condition. Cells were harvested from scaffold and plates and total RNA was extracted using Trizol, and first-strand cDNA was synthesized using Superscript II Reverse Transcriptase Kit. Quantitative PCR was performed using Power SYBR Green PCR Master Mix. Gene expression was normalized to glyceraldehyde 3-phosphate dehydrogenase (GAPDH). The relative amount of measured mRNA in samples was determined using the 2 -ΔΔ CT method where ΔΔ CT = (CT_target_ – CT_GAPDH_) sample – (CT_target_ – CT_GAPDH_) calibrator. All results were presented as mean ± (standard deviation) SD of at least n=3 experiments.

*Immunofluorescence.* The cells-laden scaffold was immersed in the OCT embedding agent and then put it in the refrigerator at -20℃. After the frozen block is made, it can be put into the constant cooling box slicer to slice the section. Successively, it can be continuously sliced to 20 μm. To stain sectioned scaffold, the section was blocked with 5% BSA in PBS-tween (PBST) and incubated with primary antibody overnight at 4 °C. To confirm albumin secretion of cells on scaffold, the tissue sections were immunofluorescent stained with primary antibodies against ALB (1:500 dilution). The stained sections were visualized with Alexa 647-conjugated secondary antibodies (abcam) and counterstained with 4′,6-diamidino-2-phenylindole (DAPI, Vector Laboratories, Burlingame, CA, U.S.A.) for nuclear staining.

*Western-blot.* The protein of cells was extracted with lysis buffer and 1% protease inhibitor cocktail. The cell lysate was centrifuged at 15,000 g for 15 min at 4℃ and the supernatant was collected. The protein of cells was adjusted in the sample buffer, and separated by sodium dodecyl sulfate polyacrylamide gel electrophoresis (SDS-PAGE) on 10% polyacrylamide gels. The separated samples were transferred onto a poly (vinylidene difluoride) (PVDF) membrane. The transferred membrane was treated by blocking at 4℃ overnight by using Blocking one. After blocking, the membrane was incubated with primary antibodies (rabbit anti-ASGPR, rabbit anti-CK18) and incubated overnight at 4℃. After incubation, the membrane was incubated with HRP conjugated secondary antibodies (1:10000 dilutions) for 2 hours at room temperature.

*PAS***.** To assess glycogen storage, sections were stained by periodic acid-Schiff (PAS, Solarbio) following the manufacturer’s instructions. Firstly, cells-loaden scaffolds were frozen and sectioned. The cells planted on the glass coverslips and frozen sections were fixed with formalin and directly immersed into distilled water. The samples were rinsed with running water for 2 minutes, and then soaked with distilled water for 2 minutes. Next, the samples were placed in the oxidizer at room temperature for 8 minutes and rinsed with running water again. After that, the samples were immersed in Schiff Reagent for 15 minutes in the dark and rinsed with running water for 10 minutes. In the end, the samples were placed in hematoxylin staining solution and stained the nuclei for 2 minutes.

*Animal experiments.* The cells-laden scaffolds were performed on the liver, spleen and omentum in nude mice for ectopic transplantation. Two weeks after transplantation, the recellularized scaffolds were taken out of the nude mice. For histological analysis, the scaffolds were embedded in OCT cryo-compound and the specimens were then sliced into 5 μm sections. The sectioned samples were stained with hematoxylin and eosin (H&E) to check the removal of cellular components. To confirm albumin secretion of cells on scaffolds, the tissue sections were stained with primary antibodies against ALB (1:500 dilution). The stained sections were visualized with Alexa 647-conjugated secondary antibodies and counterstained with DAPI for nuclear staining. The glycogen storage in the cells loaded on the scaffold was determined using the PAS kit in accordance with the manufacturer’s instructions. This study was approved by the Committee on the Ethics of Animal Experiments of the Affiliated Drum Tower Hospital of Nanjing University Medical School (Approval No. 2018010017).

*Statistical analysis.* All the data showed were normalized with the control group. All data were expressed as mean ± SD. All statistical analyses were conducted using ORIGIN software.

**Supporting Information：**

**Figure S1. The value of stress at break of natural celery and decellularized scaffold.**

**Figure S2. The detection of strain at break in natural celery and decellularized scaffold.**

**Figure S3. The fluorescent of** **ALB intensity in hiPSC-Heps adhered on 2D conventional dish, matrigel and decellularized celery scaffold.**

**Figure S4. The PAS intensity in hiPSC-Heps adhered on 2D conventional dish, matrigel and decellularized celery scaffold.**
